# Editorial: Unravelling the sarcoma microenvironment: Impact of the genomic landscape on molecular signaling, immunosuppression, and treatment resistance

**DOI:** 10.3389/fonc.2023.1180954

**Published:** 2023-03-24

**Authors:** Erin B. Dickerson, Eleanor Y. Chen, Jong Hyuk Kim

**Affiliations:** ^1^ Department of Veterinary Clinical Sciences, College of Veterinary Medicine, University of Minnesota, St. Paul, MN, United States; ^2^ Animal Cancer Care and Research Program, College of Veterinary Medicine, University of Minnesota, St. Paul, MN, United States; ^3^ Masonic Cancer Center, University of Minnesota, Minneapolis, MN, United States; ^4^ Department of Laboratory Medicine and Pathology, University of Washington, Seattle, WA, United States; ^5^ Department of Small Animal Clinical Sciences, College of Veterinary Medicine, University of Florida, Gainesville, FL, United States

**Keywords:** sarcoma, tumor microenvironment, genomic landscape, immunotherapy, tumor heterogeneity

Sarcomas encompass a large group of highly diverse tumors of mesenchymal cell origin that can arise in almost any tissue at any stage of life in the bone or soft tissues (muscle, fat, blood vessels, nerves, connective tissues and organs) (1). Their genomic landscapes may be simple or complex, where simple genomes often harbor common genomic drivers (oncogenic fusion gene, mutations, or amplifications in specific chromosomal regions), and complex genomes exhibit a highly heterogeneous array of mostly non-recurrent genetic alterations, differing intratumorally and across patients. Although the rarity of sarcomas has historically limited their study, the mutational landscape found across sarcomas represents the next challenge to scientists and clinicians as it can influence drug responses and the development of resistance, enhance immune evasion, and alter responses to immunotherapy. Outcomes for sarcoma patients will improve with the development of treatments exploiting knowledge of sarcoma genetics and communication between tumor cells and those that reside in the tumor microenvironment.

Papers in this Special Topic span a small but diverse fraction of more than 100 distinct histological types and subtypes of sarcomas that have been described (2). They address a spectrum of sarcoma-related subjects from heterogeneity, targeted treatment approaches to known mutations or amplifications, the tumor immune landscape, and the identification of drug resistance mechanisms and strategies to circumvent these processes. These studies also show how scientists, clinicians, and clinician scientists use bioinformatic and computational approaches to identify the complex mechanisms that drive sarcoma progression.

In the first of two reviews, Apfelbaum et al. comprehensively assess Ewing sarcomas, which are driven by a single fusion between a FET protein and an ETS family transcription factor, most commonly EWS::FLI1. Despite most of these tumors being driven by a single, shared driver mutation, Ewing sarcoma cells exhibit substantial intra- and intertumoral heterogeneity, attributed to the differential expression and activity of the resulting fusion protein. The authors emphasize the cell-intrinsic factors regulating fusion protein levels and studies demonstrating the impact of the tumor microenvironment in Ewing sarcoma.

In the second review, Traweek et al. assess the contributions of *MDM2* gene amplification in dedifferentiated liposarcoma (DDLPS). The overexpression of the MDM2 protein (a defining feature of DDLPS) is associated with cell cycle progression and malignant proliferation, providing the rationale for developing targeted approaches to inhibit MDM2 activity. The authors also discuss current strategies to target MDM2 and review results from recent clinical trials focused on MDM2 inhibition.

Lazcano et al. and Lv et al. present original research on the immune landscape of sarcomas and the untapped potential of immunotherapy for the treatment of these tumors. The development and clinical introduction of cancer immunotherapies has underscored the importance of the immune system in shaping, controlling, and eliminating cancer (3, 4). Despite transformative treatments for advanced melanoma, lung, and other cancers (5–9), only a fraction of sarcoma patients has benefitted from this groundbreaking approach (10). The genomic landscape plays a fundamental role in shaping the tumor immune microenvironment and determining the response to immunotherapies, as highly mutated tumors are more likely to have detectable neoantigens and drive immunity and response to immunotherapy (11). Because cancer patients with immunologically aware or “hot” tumors often have significantly better outcomes than patients with immunologically silent or “cold” tumors, revising sarcoma stratification systems to include immune parameters could transform clinical decisions and outcomes (3, 12).

Correlative studies from previous clinical trials suggested the tumor immune microenvironment of undifferentiated pleomorphic sarcoma (UPS) is associated with responses to immune checkpoint blockade and survival. Based on these results, Lazcano et al. designed a retrospective study, combining immunohistochemical (IHC) staining of markers of tumor-associated immune cells, immune checkpoints, and adenosine pathways, with unsupervised clustering of resulting IHC values. They identified three immunologically distinct clusters in primary tumors that were significantly associated with overall survival, confirming the prognostic impact of the immune microenvironment in these sarcomas. The resulting immune-based classification system could be used to identify patients more likely to respond to immune checkpoint inhibitors or other immunotherapies.

Alternatively, Lv et al. applied a multi-omics analysis to evaluate the microenvironment of osteosarcoma, identifying three molecular subgroups related to prognostic outcomes. Surprisingly, these signatures were associated with aging- and senescence-induced gene (ASIG) pathways. Further analysis showed that immune cell infiltration between groups differed significantly, and construction of risk models built upon the differentially ASIGs was found to predict patient survival and immune status. While the clinical value of their predictions needs to be confirmed experimentally, their approach provides another tool that can be used to navigate the application of immunotherapy in osteosarcoma.

Drug resistance remains a limiting factor for achieving cures in cancer patients. Identifying mechanisms of resistance and developing strategies to circumvent these processes can guide treatments to improve patient responses. Overactivation of mitogen-activated protein kinase (MAPK) signaling is nearly ubiquitous across malignant peripheral nerve sheath tumors (MPNST); however, responses to MEK inhibitor (MEKi) monotherapy remain poor. Using MEKi-resistant cell lines, Gu et al. investigated the mechanisms of MEKi resistance in MPNST, identifying compensatory pathways that limited the drug efficacy. They went on to develop a dual treatment strategy that restored the sensitivity of resistant MPNST cells to MAPK inhibition, providing a potential new treatment strategy for MPNST.

This collection of Review and Original Research articles increases what is known about the tumor microenvironment in sarcomas and how it relates to their genomic features. Continued research will be crucial in overcoming the current challenges of sarcoma therapy and providing new avenues for treating these devastating tumors.

## Author contributions

All authors listed have made substantial and intellectual contributions to the work and approved it for publication.
